# Nutritional status, growth and disease management in children with single and dual diagnosis of type 1 diabetes mellitus and coeliac disease

**DOI:** 10.1186/1471-230X-14-99

**Published:** 2014-05-28

**Authors:** Mary Mackinder, Gavin Allison, Vaios Svolos, Elaine Buchanan, Alison Johnston, Tracey Cardigan, Nicola Laird, Hazel Duncan, Karen Fraser, Christine A Edwards, Ian Craigie, Paraic McGrogan, Konstantinos Gerasimidis

**Affiliations:** 1 Human Nutrition, School of Medicine, College of Medicine, Veterinary and Life Sciences, Royal Hospital for Sick Children, University of Glasgow, G3 8SJ Glasgow, UK; 2 Department of Paediatric Diabetes, Royal Hospital for Sick Children, Glasgow, G3 8SJ Glasgow, UK; 3 Department of Paediatric Gastroenterology, Hepatology and Nutrition, Royal Hospital for Sick Children, Glasgow, G3 8SJ Glasgow, UK

**Keywords:** Coeliac, Diabetes, Glycaemic control, Growth, Nutrition

## Abstract

**Background:**

The consequences of subclinical coeliac disease (CD) in Type 1 diabetes mellitus (T1DM) remain unclear. We looked at growth, anthropometry and disease management in children with dual diagnosis (T1DM + CD) before and after CD diagnosis.

**Methods:**

Anthropometry, glycated haemoglobin (HbA1c) and IgA tissue transglutaminase (tTg) were collected prior to, and following CD diagnosis in 23 children with T1DM + CD. This group was matched for demographics, T1DM duration, age at CD diagnosis and at T1DM onset with 23 CD and 44 T1DM controls.

**Results:**

No differences in growth or anthropometry were found between children with T1DM + CD and controls at any time point. Children with T1DM + CD, had higher BMI z-score two years prior to, than at CD diagnosis (p < 0.001). BMI z-score change one year prior to CD diagnosis was lower in the T1DM + CD than the T1DM group (p = 0.009). At two years, height velocity and change in BMI z-scores were similar in all groups. No differences were observed in HbA1c between the T1DM + CD and T1DM groups before or after CD diagnosis. More children with T1DM + CD had raised tTg levels one year after CD diagnosis than CD controls (CDx to CDx + 1 yr; T1DM + CD: 100% to 71%, p = 0.180 and CD: 100% to 45%, p < 0.001); by two years there was no difference.

**Conclusions:**

No major nutrition or growth deficits were observed in children with T1DM + CD. CD diagnosis does not impact on T1DM glycaemic control. CD specific serology was comparable to children with single CD, but those with dual diagnosis may need more time to adjust to gluten free diet.

## Background

Coeliac Disease (CD) is an aberrant immunological response to ingestion of dietary gluten in individuals with genetic predisposition, causing villous atrophy, crypt hyperplasia in the mucosa of the small intestine and nutrient malabsorption. [1]. The typical clinical presentation of ‘classical’ CD includes poor linear growth and nutritional status, abdominal pain and distension, diarrhoea and iron deficiency anaemia [2]. Adherence to a lifelong gluten free diet (GFD) is the sole mainstream management approach for CD. Highly specific and sensitive serological autoimmune markers such as IgA anti-endomysial (EMA) and IgA tissue transglutaminase (tTg) are now used for routine screening of CD; enabling the identification of ‘silent’ and ‘atypical’ forms which do not express the ‘classical’ features of symptomatic CD [3].

Individuals with Type 1 diabetes mellitus (T1DM) are at increased risk of developing CD [4]. Genetic predisposition [5], young age at T1DM onset [6], female gender [7] and early introduction of gluten in the infant’s diet [8,9] have been associated with an increased risk of development of CD in people with T1DM. Despite the substantial occurrence of CD in people with T1DM (2-12%), routine serological screening of those at increased risk remains controversial [10].

A review of the recent primary literature demonstrated that in those health services that practise routine serological screening for CD in people with T1DM, anthropometry and growth parameters were reported to be within the normal reference values at the time of CD diagnosis [7,11-14] (Additional file 1: Table S1). However children with dual diagnosis did not grow as well as their T1DM peers, [7,15] presenting with greater deficits in weight [7,15,16], height [16,17] and BMI z-scores [15,16]. In contrast, in two studies in centres without regular screening, growth and nutritional status deficits were more pronounced in children with T1DM + CD [16,17] (Additional file 1: Table S1).

It has been suggested that the destruction of the small bowel mucosal architecture in those with T1DM but undiagnosed CD, causes malabsorption of nutrients which may cause reduction in glycated haemoglobin A1c (HbA1c) levels [14,16,18], lower insulin requirements [11,12] and increase the frequency of self-reported severe hypoglycaemic episodes (Additional file 1: Table S1). Yet the evidence remains inconsistent and other studies have reported no difference in HbA1c levels [7,12,13,17], nor in the number of severe hypoglycaemic episodes [7,13-17] (Additional file 1: Table S1). Inconsistency of study results (Additional file 1: Table S1) may be caused by differences in cohort size, study design, absent or poorly matched control groups [11,16], lack of data on compliance to GFD [7,11,14,15,19], inaccurate self-reporting of glycaemic episodes, varied screening practices, availability of dietetic support among health centres and the duration of CD diagnosis delay (Additional file 1: Table S1). Thus far, no study looked at the impact of dual diagnosis on the management of CD and compliance with gluten free diet (GFD).

In the current study we determined the pattern of growth, anthropometry and disease management of CD and T1DM prior to and after the diagnosis and treatment of CD in screen detected and endoscopically diagnosed children with T1DM + CD and compared these against precisely matched control groups of children with single diagnosis of T1DM or CD.

## Methods

The present study included children with T1DM, CD or dual diagnosis (T1DM + CD) regularly attending the relevant outpatient clinics at the Royal Hospital for Sick Children, Glasgow, UK. Data were extracted from two prospective clinical databases held by the local departments of Paediatric Gastroenterology, Hepatology and Nutrition and Paediatric Diabetes. At the time of the study, the T1DM database contained 1203 people with T1DM treated locally from 2000 to 2012. Individuals were reviewed routinely every 3–4 months. Information on growth parameters (e.g. height) and nutritional status (e.g. weight and BMI), and HbA1c, as an index of glycaemic control, were recorded in the database at each clinical assessment. From 2002, T1DM children were screened at diagnosis and at 2 year intervals for specific CD serological markers, anti-endomysial antibodies (EMA) and from 2003 tTg, or IgG EMA in the case of IgA deficiency. Those with positive serology underwent endoscopic investigations to confirm diagnosis. Those diagnosed with CD based on the Marsh criteria were advised to follow a GFD.

From this cohort, 45 children with T1DM were diagnosed with CD. For the purposes of this study we selected all children with a T1DM history longer than 2 years prior to CD diagnosis to eliminate the characteristic weight faltering prior to T1DM diagnosis and weight gain associated with initiation of exogenous insulin. The selected participants had no other chronic medical condition, and had anthropometric data available for two years prior and two years post CD diagnosis. In total, there were 23 children with T1DM + CD who fulfilled these criteria. These were matched (1:2 since there were at least twice as many children) for age, sex, age at T1DM onset and duration of T1DM to a group of children (n = 44) with only T1DM. Similarly the T1DM + CD group were matched (1:1) for age, sex, age at CD diagnosis to a control group of CD (n = 23) obtained from the gastroenterology clinical database of 293 CD children under the care of the local clinical team from 1997 to 2012. Children with CD were seen in the coeliac outpatients clinic at 3, 6 and 12 months after initial diagnosis and annually thereafter. Information on growth parameters (e.g. height), and nutritional status (e.g. weight and BMI) and tTg as a proxy of disease management and compliance with GFD, were collected during each hospital visit.

### Data handling

**Figure 1:**
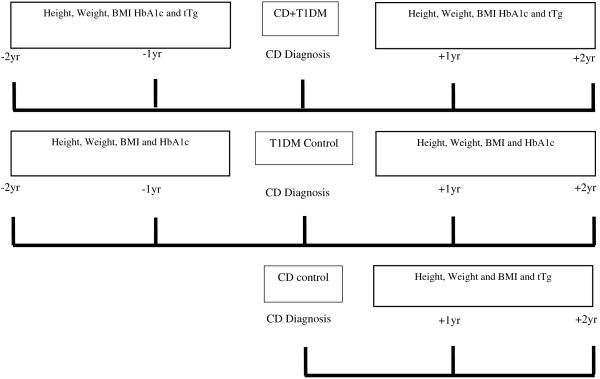
Study design with data collected at each time point of the follow up.

The children with T1DM + CD were considered the primary comparison group and anthropometric measurements were taken at CD diagnosis and one year and two years prior to and post diagnosis (Figure 1). Both the CD and T1DM children were matched to the T1DM + CD group at each time point (Figure 1). Height, weight and BMI z-scores were calculated using the 1990 UK reference data [20]. Growth velocity and changes in nutritional status were computed as the difference (∆) in height z-scores and BMI z-scores between CD diagnosis and the time points one and two years before and after diagnosis. BMI was classified as ‘Thin’ (BMI ≤ −2SD), ‘Normal weight’ (−2SD < BMI < 2SD) and ‘Obese’ (BMI ≥ 2SD). Similarly participant height was classified as ‘Short’ or “Normal” if this fell below or above −2 SD respectively. In CD children, disease activity and compliance with gluten free diet, was classified based on the tTG levels [‘Normal’ (tTg < 7 U/ml) and ‘Abnormal’ (tTg ≥ 7 U/ml) [21]]. Likewise in children with T1DM, metabolic control was classed into ‘Optimal’ (HbA1c <7.5%) and ‘Suboptimal’ (HbA1c ≥7.5%)] at each time point. In addition for patients with diabetes the median HbA1c (%) was calculated from the six clinical assessments before and after CD diagnosis to serve as a long term proxy index of metabolic control T1DM.

### Statistical analysis

Chi-squared and Fisher’s exact test were used to assess differences in qualitative data (e.g. frequency of abnormal HbA1c between people with T1DM + CD and T1DM). For continuous non-parametric data, differences between groups were tested with the Kruskal-Wallis and Mann–Whitney U tests. Comparison of means of normally distributed data was carried out with 2-sample t-test. One sample t-test was used to assess deviations of height z-scores from the national reference data (UK 1990) and paired t-tests and 1-sample Wilcoxon tests to assess differences within a single group at different time points. Pearson correlation was used to assess the linear association between continuous variables. P-value <0.05 were considered statistically significant. Minitab (version 16, UK) was used for data analysis.

### Ethics

Caldicott Guardian Approval from the National Health Service was granted to carry out this study.

## Results

### Subject characteristics

From a total of 1203 T1DM children who received care at the local hospital 45 (3.7%) were also diagnosed with CD. Those with dual diagnosis (T1DM + CD) were younger at diabetes onset [mean ± SD, years: (5.4 ± 0.7 vs 7.4 ± 6.4; p = 0.011)] than the remaining cohort of T1DM children (n = 1158). Likewise children with T1DM + CD were diagnosed with CD at an older age [mean ± SD, years: (10.8 ± 4.3 vs 7.3 ± 2.8, p < 0.001)] than the entire cohort of children with CD (n = 293).

The 23 children with T1DM + CD who fulfilled the inclusion criteria of the study were representative of all the 45 T1DM + CD children (22 were excluded: 2 secondary concomitant illness, 4 recently diagnosed and 16 missing data), with no significant differences regarding sex and age at diagnosis of CD or T1DM (p = 0.853, p = 0.239 and p = 0.790 respectively). As expected there were no significant differences in sex [Males; T1DM + CD: 11/23 (48%); CD: 11/23 (48%); T1DM: 21/44 (48%)], age at T1DM diagnosis (mean ± SD; T1DM + CD: 5.3 ± 3.4; T1DM: 5.0 ± 3.1; years), age at CD diagnosis (mean ± SD; T1DM + CD: 10.7 ± 2.8; CD: 10.7 ± 2.9; years) and duration of T1DM at CD diagnosis (mean ± SD; T1DM + CD: 5.4 ± 3.2; T1DM: 5.6 ± 3.2; years), between the groups (Table 1).

**Table 1:** Subject characteristics of children with coeliac disease (CD), type 1 diabetes mellitus (T1DM) and dual diagnosis (T1DM + CD)

	**T1DM + CD**	**T1DM**	**CD**
**Number of participants (n)**	23	44	23
**Gender (M/F: n/percentage)**	M: 11/48%	M: 21/48%	M: 11/48%
	F: 12/52%	F: 23/52%	F: 12/52%
**Age at T1DM diagnosis (years; mean ± SD)**	5.3 ± 3.4	5 ± 3.1	n/a
**Age at CD diagnosis (years; mean ± SD)**	10.7 ± 2.8	n/a	10.7 ± 2.9
**Duration of T1DM on CD diagnosis (years; mean ± SD)**	5.4 ± 3.2	5.6 ± 3.2	n/a

Coeliac Disease (CD), Type 1 Diabetes Mellitus (T1DM).

### Growth and nutritional status

#### Comparison against the UK 1990 reference data

In children with T1DM + CD, height, weight and BMI z-scores were not significantly different to the UK reference values at any time point of the study (Table 2). Children with CD had significantly lower height z-scores at CD diagnosis (CDx), at one (CDx + 1 yr) and two (CDx + 2 yrs) years post diagnosis compared to the UK 1990 reference population data [Height z-score, mean ± SD: (CDx: −0.72 ± 0.97, p = 0.002; CD + 1 yr: −0.63 ± 1.19, p = 0.023 and CDx + 2 yrs:-0.32 ± 0.76, p = 0.044)] (Table 2). In the same group, BMI and weight z-scores did not deviate from the national norms at any time point of the study (Table 2). Children with T1DM had no differences in BMI or height z-scores from the reference population, however they were heavier for their age at the majority of time points [weight z-score, mean ± SD: (CDx-1 yrs: 0.26 ± 0.83, p = 0.05; CDx: 0.36 ± 0.85, p = 0.008; CDx + 1 yrs: 0.30 + 0.85, p = 0.027; CDx + 2 yrs: 0.29 ± 0.81, p = 0.032)] (Table 2).

**Table 2:** Nutritional status and growth of children with coeliac disease (CD), type 1 diabetes mellitus (T1DM) and dual diagnosis (T1DM + CD)

Mean ± SD*	**Condition**	**CDx-2 yrs**	**CDx-1 yrs**	**CDx**	**CDx + 1 yr**	**CDx + 2 yrs**
**BMI z-score, (SD)**	*T1DM + CD*	0.14 ± 0.95^2^	0.14 ± 0.90	−0.03 ± 1.01	−0.04 ± 0.99	0.07 ± 1.02
	*CD*	n/a	n/a	0.09 ± 1.09	0.13 ± 1.23	0.27 ± 1.18
	*T1DM*	0.49 ± 1.02	0.26 ± 0.95	0.39 ± 0.92	0.37 ± 0.9	0.27 ± 0.92
**BMI classification, *Th/N/Ob *** **(n)**	*T1DM + CD*	0/23/0	0/23/0	1/21/0	1/22/0	1/17/0
	*CD*	n/a	n/a	1/21/1	0/21/1	1/13/3
	*T1DM*	0/40/3	0/43/1	0/43/1	0/41/1	0/38/1
						
**Height z-score, (SD)**	*T1DM + CD*	−0.44 ± 1.38	−0.36 ± 1.26	−0.26 ± 1.21	−0.32 ± 1.10	−0.38 ± 1.61
	*CD*	n/a	n/a	−0.72 ± 0.97^1^	−0.63 ± 1.19^1^	−0.32 ± 0.76^1^
	*T1DM*	−0.11 ± 0.78	0.03 ± 0.72	0.05 ± 0.77	−0.05 ± 0.72	0.07 ± 0.69
**Height classification *Short/Normal *** **(n)**	*T1DM + CD*	2/21	2/20	2/21	2/21	3/15
	*CD*	n/a	n/a	3/20	1/21	0/16
	*T1DM*	0/43	0/44	0/44	0/44	0/39
						
**Weight z-score, (SD)**	*T1DM + CD*	−0.21 ± 1.30	−0.12 ± 1.26	−0.19 ± 1.26	−0.2 ± 1.04	−0.15 ± 1.48
	*CD*	n/a	n/a	−0.31 + 0.83	−0.22 ± 0.89	0.06 ± 1.02
	*T1DM*	0.33 ± 0.85	0.26 ± 0.83^1^	0.36 ± 0.85^1^	0.30 + 0.85^1^	0.29 ± 0.81^1^
						

Coeliac Disease (CD), Type 1 Diabetes Mellitus (T1DM), CDx: Coeliac Disease diagnosis; CDx + 1: One year post Coeliac Disease diagnosis; CDx + 2: Two years post Coeliac Disease diagnosis; CDx-1: One year prior Coeliac Disease diagnosis; CDx-2y: Two years prior Coeliac Disease diagnosis; HbA1c: Glycated haemoglobin A1c; tTG: Tissue Transglutaminase; BMI: Body mass index; Th/N/Ob: number of thin/normal BMI/obese participants; ^1^p < 0.05 to UK 1990 reference data; ^2^p < 0.001 between CDx and each time points; *unless otherwise is specified; n/a: data for CD were not available prior to disease diagnosis.

#### Comparison between groups at different time points

Mean measurements of height, weight and BMI z-scores were similar between the T1DM + CD and CD control group at all the time points. Similarly, there were no significant differences in height or BMI z-scores between the T1DM + CD and T1DM controls before or after CD diagnosis (Table 2). In the latter groups, children with T1DM + CD tended to be lighter (weight z-score) than those with T1DM two years prior to diagnosis, at CD diagnosis and one year after CD diagnosis although the mean difference did not reach statistical significance [T1DM + CD vs T1DM: weight z-score, mean ± SD: (CDx-2 yrs: −0.21 ± 1.30 vs 0.33 ± 0.85, p = 0.08, CDx: −0.19 ± 1.26 vs 0.36 ± 0.85, p = 0.07 and CDx + 1 yr −0.19 ± 1.26 vs 0.36 ± 0.85, p = 0.06)] (Table 2).

Two years prior to CD diagnosis, the change in BMI z-score (∆BMI z-score) did not differ between T1DM children and those with T1DM + CD. However at one year prior to CD diagnosis, the ∆BMI z-score was negative and significantly lower in the T1DM + CD than the T1DM children whose mean ∆BMI z-score increased [∆BMI z-score, mean ± SD: T1DM + CD: −0.17 ± 0.4 vs T1DM: 0.14 ± 0.5, p = 0.009)]. One and two years post-diagnosis, there were no significant differences in nutritional status (∆BMI z-score) and growth velocity (∆Height z-score) between any of the three groups.

#### Changes within each group before and after CD diagnosis

In the children with T1DM + CD, BMI z-score was significantly higher two years prior to CD diagnosis than at CD diagnosis [BMI z-score, mean ± SD: (CDx-2 yrs: 0.14 ± 0.95 vs CDx: −0.03 ± 1.62, p < 0.001)]. This was not observed in the T1DM controls (Table 2). Compared to CD diagnosis, BMI z-score did not change at any of the follow up time points (Table 2). There were no differences in height or weight z-scores in any group before and after CD diagnosis (Table 2).

#### Glycaemic control between T1DM and T1DM + CD

**Figure 2:**
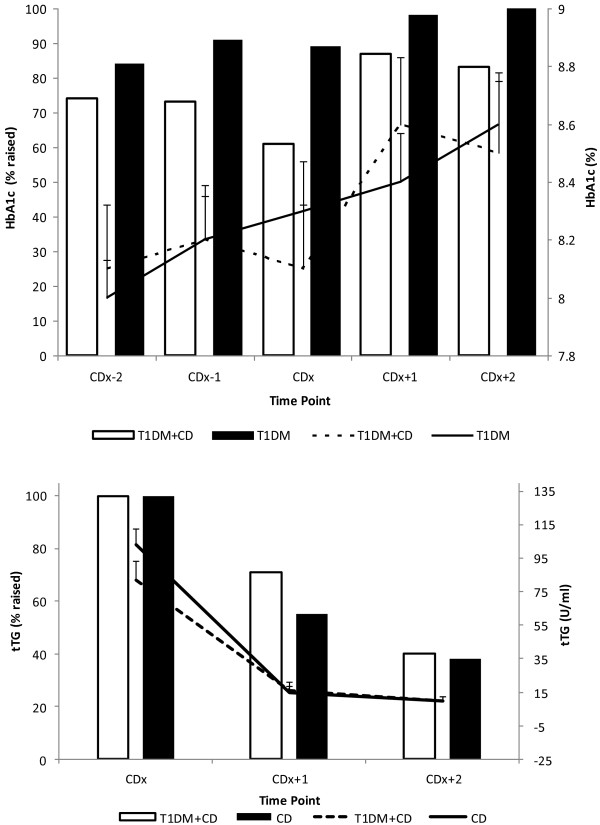
**Levels (line) and percentage (bars) of participants with raised HbA1c and tTG in children with Coeliac Disease (CD), Type 1 Diabetes Mellitus (T1DM) and dual diagnosis (T1DM + DM).** Top panel: Left axis displays percentage (bars) of participants with raised HbA1c (>7.5%); right axis displays mean (lines) HbA1c levels (%) with error (SEM) bars. Bottom panel: Left axis displays percentage (bars) of participants with raised tTG (>7.0 U/ml); right axis displays mean (lines) tTG levels (U/ml) with error (SEM) bars. CD: Coeliac Disease; T1DM: Type 1 Diabetes Mellitus; T1DM + DM: Type 1 Diabetes Mellitus and Coeliac Disease; tTG: Tissue Transglutaminase; HbA1c: Glycated haemoglobin A1c; CDx: Coeliac Disease diagnosis; CDx + 1: One year post Coeliac Disease diagnosis; CDx + 2: Two years post Coeliac Disease diagnosis; CDx-1: One year prior Coeliac Disease diagnosis; CDx-2y: Two years prior Coeliac Disease diagnosis.

In both T1DM + CD and T1DM groups, HbA1c (%) significantly increased after CD diagnosis (baseline) [HbA1c, mean ± SD: (CDx to CDx + 2 yrs, T1DM + CD: 8.1 ± 1.1% to 8.5 ± 1.1%, p < 0.05 and T1DM: 8.3 ± 1.1% to 8.6 ± 1.1%, p < 0.05)] (Figure 2). Mean HbA1c (%) did not differ between the T1DM + CD and T1DM and neither did the proportion of children with raised HbA1c levels (≥7.5% HbA1c) at any time point (Figure 2). Similarly there was no significant difference between T1DM + CD and T1DM groups regarding the median of all cumulative HbA1c measurements collected preceding (n = 6) or following (n = 6) CD diagnosis [Mean ± SD HbA1c %, T1DM + CD vs T1DM: (CDx-2 yrs to CDx: 8.2 ± 0.8 vs 8.2 ± 0.8, p = 0.917 and CDx to CDx + 2 yrs: 8.4 ± 0.9 vs 8.4 ± 1.0; p = 0.923)]. In the T1DM + CD, there were no differences in the percentage of children classified with raised HbA1c levels before and after CD diagnosis. No significant correlations were observed between HbA1c levels and tTg concentrations at CD diagnosis or one or two years after CD diagnosis (Pearson correlation; CDx: r = −0.214, p = 0.409, CDx + 1 yr: r = 0.370, p = 0.193, CDx + 2 yrs: r = 0.167, p = 0.553).

#### Compliance to a GFD in CD and T1DM + CD groups

Mean tTg concentrations did not differ between the children with T1DM + CD and CD at diagnosis and during follow up (Figure 2). Compared to CD diagnosis, plasma tTg concentrations significantly decreased one and two years after treatment with GFD in both groups (T1DM + CD and CD) (p < 0.001) (Figure 2) indicating compliance to the GFD. However a significantly higher proportion of children in the T1DM + CD group had raised tTg levels (>7.0 U/ml) one year after CD diagnosis compared with the CD controls (CDx to CDx + 1 yr; T1DM + CD: 100% to 71%, p = 0.18 and CD: 100% to 45%, p < 0.001) (Figure 2). At two years post diagnosis the difference in the percentage of children with abnormal tTg levels was no longer different (T1DM + CD: 40% vs CD: 38%, p = 0.943) and both groups had significantly fewer children with raised levels compared with CD diagnosis (p < 0.001) (Figure 2).

## Discussion

This study characterised the growth, nutritional status and management of CD and T1DM in children with dual diagnoses before and after the diagnosis of CD by comparison with a matched cohort of CD and T1DM controls. Such aspects make this study unique and overcome the limitations of previous research [7,11,12,14,16] (Additional file 1: Table S1). In summary, the findings of this study suggest that the dual diagnoses of CD and T1DM do not affect the nutritional status, growth and disease management of either condition. This is in accordance with previous studies which reported no difference in the growth patterns of children with T1DM + CD at CD diagnosis compared with their peers with T1DM (14–16) but contrary to others, who found deterioration of linear growth [7,17], weight [7,17,22,23] and BMI z-scores [23] both at and post CD diagnosis (Additional file 1: Table S1). Inconsistency between studies may be explained by differences in screening practices between health centres, delayed diagnosis of CD and variations in sample size or sample selection bias by comparing groups of participants with different characteristics (Additional file 1: Table S1). Indeed, studies that reported similar results to the current study were located in centres which had annual screening programs for CD in people with T1DM. In studies where CD screening was not routine practice, height and weight z-scores deficits were observed in children with T1DM + CD compared with T1DM controls [16,17].

Weight gain is a recognised feature in individuals with T1DM on intensive insulin therapy [24]. T1DM is often characterised by weight loss prior to diagnosis of diabetes and weight gain following initiation of insulin therapy. This is a confounder in previous studies that did not account for fluctuations in growth at the start of insulin therapy [11,13,17]. In the current study, all children with T1DM and T1DM + CD were specified to have been diagnosed with T1DM diagnosis more than two years prior to CD diagnosis to eliminate the remission phase after T1DM diagnosis. Moreover as the T1DM control group was also matched for age at T1DM diagnosis and duration of T1DM at CD onset (baseline) any influence would be reflected evenly in both groups.

Our results are consistent with many studies reporting no significant difference in HbA1c levels between individuals with T1DM and T1DM + CD before or after the diagnosis of CD [12,13]. However, other studies have reported lower HbA1c levels [14,16] or raised HbA1c levels [25], along with a reduction in insulin requirements and an increase in the number of hypoglycaemic episodes in the period prior to CD diagnosis [11,12]. This was attributed by the authors to a possible destruction of the intestinal mucosa impinging nutrient absorption [14]. In the current study, HbA1c levels were raised significantly one and two years post CDx in the T1DM + CD children, however a similar increase was observed in those with T1DM which suggests that this is a feature of T1DM disease progression rather than a result of CD diagnosis. This may be due to adolescents presenting poorer adherence to insulin therapy [26] and a large proportion of T1DM + CD are diagnosed during this age.The absence of significant fluctuations in HbA1c levels in the children with T1DM + CD in our study may be a result of the regular screening practices, predisposing to the earlier diagnosis of CD, but also due to the high level of dietetic supervision that these children receive in our centre.

Strict compliance to a GFD is associated with mucosal histology restoration and improved nutritional status in people with CD but adherence is difficult to assess accurately and has been frequently over-looked in studies [7,11,14,15]. In this study a significantly lower proportion of T1DM + CD children presented with normal tTg levels (<7.0 U/ml) one year post CD diagnosis than those with CD; by two years the majority of children in both groups had tTg levels within the normal values. This may reflect that people with T1DM + CD need more time to adapt to the challenges and dietary management of both conditions particularly as the large majority were diagnosed during adolescence, when adherence to GFD is reported between 25-60% [18,19]. On the other hand, active on-going symptoms in those with CD may encourage adherence to a GFD making the dietary changes more acceptable. Moreover, individuals with T1DM + CD identified by serological testing with “silent” CD have an absence of overt clinical symptoms, therefore the GFD may be perceived as of secondary priority to the management of diabetes and its associated complications.

One year prior to CD diagnosis, the mean change in BMI z-scores in the children with T1DM tended to increase but in those with T1DM + CD, the average change in BMI z-scores were significantly lower and negative. These differences in nutritional status change may indicate the onset of CD before diagnosis, which might have affected nutrient absorption and subsequently nutritional status. This pattern was reversed within two years after the introduction of GFD, supporting our speculations. Moreover it is possible that further deterioration might have been prevented by routine screening and early detection of CD in these people, limiting the time CD would remain undiagnosed. In children with CD it is well established that the longer active CD remains undiagnosed and the older the age before this is diagnosed are both associated with complications like gastrointestinal malignancy [17] and osteoporosis [27,28]. Yet in T1DM the long-term effects of sub-clinical CD are not well investigated and remain grossly unknown. It has been speculated that undetected and untreated CD may compound the effects of T1DM and increase the risk of the developing of CD associated complications, delaying the benefits to nutritional status and psychological wellbeing associated with GFD introduction [1,29].

The retrospective design of this study is an inherent limitation as often leads to incomplete datasets and sample selection bias Moreover, data on insulin requirements and number of hypoglycaemic episodes, which would reflect a better assessment of T1DM management, were not available.

High quality longitudinal studies are required to understand the long-term outcomes of T1DM + CD. Due to lack of evidence the National Institute of Clinical Excellence (NICE) in the UK revised its guidelines reducing routine screening to a single screen at T1DM diagnosis [30]. Evidence from this study suggests that regular screening may have prevented the further decline in nutritional status and tackled deterioration of growth velocity in people with silent T1DM + CD.

## Conclusions

The early diagnosis of CD and initiation of a GFD under regular dietetic supervision may prevent further deterioration in the nutritional status of children with T1DM + CD and may also reduce the prospect of CD complications without having any evident impact on T1DM control. In centres where routine screening for CD does not occur, clinicians should remain vigilant for these clinical features and test for it accordingly.

## Abbreviations

CD: Coeliac disease; T1DM: Type 1 Diabetes Mellitus; T1DM + DM: Type 1 Diabetes Mellitus and Coeliac Disease.

## Competing interests

The authors declare no competing interests relevant to this study.

## Authors’ contributions

MM, GA, VS, EB, AJ, TG, NL, HD, KF contributed to the data collection; MM & KG carried out statistical analysis; MM, VS, IC, PM, CAE, KG interpreted the data; MM & KG drafted the paper; IC, PM, CAE, KG coordinated research activities and supervised the student (MM). All authors read and approved the final manuscript.

## Pre-publication history

The pre-publication history for this paper can be accessed here:

http://www.biomedcentral.com/1471-230X/14/99/prepub

## Supplementary Material

Additional file 1 Evidence table of recent studies exploring the impact of dual diagnosis of Type 1 diabetes and coeliac disease on anthropometry, growth and disease management. Click here for file
