# Supplementary material for: Nutritional status, growth and disease management in children with single and dual diagnosis of type 1 diabetes mellitus and coeliac disease
Source: BMC Gastroenterol. 2014 May 28;14:99. doi: 10.1186/1471-230X-14-99 (PMC4046848; doi:10.1186/1471-230X-14-99)
Supplement: Additional file 1 — Evidence table of recent studies exploring the impact of dual diagnosis of Type 1 diabetes and coeliac disease on anthropometry, growth and disease management. [file 1471-230X-14-99-S1.pdf]

Study	Subjects	Serological screening for CD	Anthropometrics at CDx	Anthropometrics at follow up (FU).	Diabetes management and glycaemic control	Compliance to GFD	Comments
[1] Germany; Multicentre, retrospective study	41,951 T1DM (0.1 to 20y); 411 T1DM+CD.	Variable (EMA and tTg)	T1DM+CD weight SDS and height SDS lower than T1DM (0.20 vs 0.43, p<0.001 and -0.28 vs -0.03, p<0.01). BMI SDS ns.	5y FU weight SDS lower in T1DM+CD than T1DM (0.23±0.93 vs 0.43±0.90, p<0.02). Height SDS lower (0.08±10.07 vs 0.15±0.95, p<0.05). BMI SDS ns.	HbA1c (%) levels at CDx or post CDx ns between groups. T1DM+CD: HbA1c (%) levels ns at CDx and FU. T1DM+CD vs T1DM: Nº hypo ns		
[2] USA; Cross-sectional, prospective	71 T1DM with tTg +ve and 63 tTg –ve T1DM controls. (2-18y)	Routine screening for IgA tTg	tTg +ve: weight SDS lower than tTg –ve (0.25±0.12 vs 0.65±0.14, p=0.03), BMI and height SDS ns.	Weight SDS lower in tTg +ve group than tTg –ve at 1y and 2y FU (0.27±0.12 vs 0.67±14, p=0.4 and 0.18±0.12 vs 0.66±0.15, p=0.01). At 2y FU BMI SDS was lower in tTg +ve group than tTg –ve group (0.28±0.17 vs 1.07±0.20, p=0.003).	CDx: IR lower in tTg +ve than tTg –ve group (0.08±0.1 vs 1.2±0.2, p=0.03). HbA1c (%) levels and the No hypo ns in tTg +ve on a GFD and those on a regular diet at CDx, 12m or 24m FU.	Adherence to a GFD was suboptimal	Baseline height, weight and BMI SDS not low. .
[3] Ireland; Retrospective study, 2000-2007	468 T1DM, 33 +ve serology. 23 CD biopsy +ve.	Annual Screening (AGA and EMA, tTg after 2002)		Weight SDS, BMI SDS and height SDS ns 12m before or after CDx.	T1DM+CD: Nº hypo lower at CDx-1y than CDx+1y (9% vs 36, p<0.07). IR higher at CDx+1y than CDx-1y (0.88±0.33 vs 1.1±0.29 units/kg/day, p<0.005). HbA1c (%) CDx +/-1y ns.	Serological compliance limited.	No matched controls.
[4] UK; Cross-sectional Multicentre	49 T1DM+CD. Matched T1DM controls.	Annual screening, AGA, EMA, and tTg	T1DM+CD vs T1DM: Weight, height and BMI SDS ns.	Weight, height and BMI SDS ns.	T1DM+CD: HbAc1 (%) lower than T1DM (8.4±1.3% vs 8.7±1.5%, p=0.01) at CDx. HbA1c (%) increase after CDx (p=0.04). T1DM+CD vs T1DM IR ns.		HbA1c increase post CDx, may indicate intestinal recovery
[5] USA; Prospective, Cross-sectional	35 biopsy CD. (2-18y) Matched to 63 T1DM tTg –ve T1DM.	Routine screening for IgA tTg	T1DM+CD lower BMI SDS than T1DM (0.36±0.87 vs 0.68±0.67, p=0.05). Height, weight SDS ns. tTg +ve: weight and BMI SDS lower than tTg –ve (0.29±1.0 vs 0.68±0.84, p=0.024 and 0.34±0.9 vs 0.75±0.76, p=0.005).		T1DM+CD vs T1DM: HbA1c (%) and IR at CDx ns. Between CDx and CDx-2y ns.		Only 20% of tTg +ve patients were on GFD for varying durations may affect results.
[6] Denmark; Prospective	269 T1DM (9.4y) (28/269 biopsy CD)	Study entry only, no prior screening.	Weight SDS and height SDS lower in T1DM+CD than T1DM (0.1 vs 0.5, p=0.002), (-0.65 vs 0.4, p<0.001).	T1DM+CD weight SDS increased from CDx to 12, 18 and 24m (p=0.002), Height SDS increase from CDx to 24m (p=0.073), excluding >14yrs (p=0.036).	T1DM+CD: HbA1c levels at CDx compared post CDx ns (p=0.3).	tTg measured. Compliance not classified or assessed	Significant lower height at CDx may be due to lack of screening in this population

[7] Austria/Hungary; Multicentre prospective	98 T1DM with +ve EMA, 74 T1DM+ biopsy CD. 195 T1DM controls. Paediatrics.	Annual screening, EMA.	T1DM+CD vs T1DM: BMI SDS ns.	T1DM+CD vs T1DM: BMI SDS, height SDS and weight SDS ns. BMI SDS:higher in compliant than non-compliant, in boys (p<0.02), in girls (p=<0.01) in one year. Height SDS ns.	T1DM+CD vs T1DM: IR and N° hypo ns.	EMA yearly. 33 compliant (EMA-ve), 25 non-compliant (EMA+ve).	
[8] Germany; Prospective	12 T1DM+CD. (1.4-25y).	Study only; IgA, AGA, EmA		Compliant: T1DM+CD height SDS increase at FU (p=0.03), non-compliant ns	HbA1c (%) lower in compliant than non-compliant T1DM+CD at CDx (8.0±0.5% vs 7.3±0.7%, p=0.05).	Clinical assessment and serology. 5/9 compliant	No controls. Small number.
[9] Australia; Prospective	21 T1DM+CD, 42 matched T1DM(1.6-12.9y).	No screening. Patients identified when symptomatic.	T1DM+CD vs T1DM: weight SDS, height SDS and BMI SDS ns,	T1DM+CD: CDx to CDx+12m: weight SDS increase (0.43±1.27 to 0.76±1.27, p=0.049), BMI SDS increase (0.44±1.16 to 0.76±0.89, p=0.015). Height SDS ns.	CDx: IR lower in T1DM+CD than T1DM (Mean difference -0.24 (-0.48, 0.005), p=0.054). IR increased in FU.T1DM+CD vs T1DM: HbA1c (%) ns at CDx and FU.	One telephone questionnaire to establish adherence (taken 3-112 m in FU).	No blood markers of CD activity.
[10] Germany + Austria; Multicentre, retrospective	127 T1DM+CD. 18,470 T1DM and 1,199 T1DM+ CD	Variable between centres.	T1DM+CD lower height SDS than T1DM (-0.49±1.1 vs -0.06±1.0, p<0.001) and lower BMI SDS (0.2±0.8 vs 0.5±0.9, p<0.001).	T1DM+CD vs T1DM height SDS ns. Children <11yrs contributed most to lost in height of T1DM+CD.	T1DM+CD vs T1DM: IR and N° of IR ns. HbA1c (%) lower in T1DM+CD than T1DM (8.1±1.85% vs 8.8±2.45%, p<0.001) at CDx + FU.	No	Patients not matched for age at CDx.
[11] Italy;Retrospective	434 T1DM, 21/434 T1DM+CD. (1.8-22yrs).	Annual IgA, IgG EMA and AGA.	T1DM+CD vs T1DM: height SDS, weight SDS and BMI SDS ns.	T1DM+CD vs T1DM: height SDS, weight SDS and BMI SDS ns.	T1DM+CD vs T1DM: increased No hypo from prior to CDx vs post CDx (4.5±4 vs 2±2.2 episodes/m. p=0.01). IR decreased from CDx-18m to CDx (0.6±0.2 vs 0.9±0.3, p=0.05). HbA1c (%) ns.	EMA/AGA, Compliance not classified or assessed.	
[12] Australia; Retrospective	20 T1DM+CD, 20 matched T1DM controls (<18y).	Annual screening	T1DM+CD vs T1DM: height SDS weight SDS and BMI SDS ns.	T1DM+CD vs T1DM: height SDS, weight SDS and BMI SDS ns. T1DM+CD: weight SDS increase from CDx to FU (-0.12±0.67 vs 0.20±0.84, p<0.05).	T1DM+CD: HbA1c (%) associated with compliance ns.	Assessed by dietary records. IgA AGA for 12/20.	Sub groups of compliance very small.

Type one Diabetes Mellitus (T1DM), Coeliac Disease (CD), Type 1 diabetes Mellitus and coeliac disease (T1DM+CD), Coeliac disease diagnosis (CDx), Gluten Free Diet (GFD), Glycosylated Haemoglobin A1c (HbA1c), Tissue transglutaminase (tTg), Standard Deviation Score (SDS), Mean Difference (MD) with 95% confidence intervals in (), Immunogloulin A (IgA), Immunoglobulin G (IgG), IGA and IgGgliadin antibodies (AGA), IgA endomysiumantibodes (EmA). Follow up (FU), Non-significant difference (ns). Insulin Requirement (IR), Hypoglycaemic episodes (hypo), year (y), months (m)

References for supplementary Table 1

1. Frohlich-Reiterer EE, Kaspers S, Hofer S, Schober E, Kordonouri O, Pozza SB, Holl RW: **Anthropometry, metabolic control, and follow-up in children and adolescents with type 1 diabetes mellitus and biopsy-proven celiac disease**. *JPediatr* 2011, **158**(4):589-593.
2. Simmons JH, Klingensmith GJ, McFann K, Rewers M, Ide LM, Taki I, Liu E, Hoffenberg EJ: **Celiac autoimmunity in children with type 1 diabetes: a two-year follow-up**. *JPediatr* 2011, **158**(2):276-281.
3. Abid N, McGlone O, Cardwell C, McCallion W, Carson D: **Clinical and metabolic effects of gluten free diet in children with type 1 diabetes and coeliac disease**. *PediatrDiabetes* 2011, **12**(4 Pt 1):322-325.
4. Sun S, Puttha R, Ghezaiel S, Skae M, Cooper C, Amin R: **The effect of biopsy-positive silent coeliac disease and treatment with a gluten-free diet on growth and glycaemic control in children with Type 1 diabetes**. *DiabetMed* 2009, **26**(12):1250-1254.
5. Simmons JH, Klingensmith GJ, McFann K, Rewers M, Taylor J, Emery LM, Taki I, Vanyi S, Liu E, Hoffenberg EJ: **Impact of celiac autoimmunity on children with type 1 diabetes**. *JPediatr* 2007, **150**(5):461-466.
6. Hansen D, Brock-Jacobsen B, Lund E, Bjorn C, Hansen LP, Nielsen C, Fenger C, Lillevang ST, Husby S: **Clinical benefit of a gluten-free diet in type 1 diabetic children with screening-detected celiac disease - A population-based screening study with 2 years' follow-up**. *Diabetes Care* 2006, **29**(11):2452-2456.
7. Rami B, Sumnik Z, Schober E, Waldhor T, Battelino T, Bratanic N, Kurti K, Lebl J, Limbert C, Madacsy L *et al*: **Screening detected celiac disease in children with type 1 diabetes mellitus: Effect on the clinical course - (A case control study)**. *Journal of Pediatric Gastroenterology and Nutrition* 2005, **41**(3):317-321.
8. Sanchez-Albisua I, Wolf J, Neu A, Geiger H, Wascher I, Stern M: **Coeliac disease in children with Type 1 diabetes mellitus: the effect of the gluten-free diet**. *DiabetMed* 2005, **22**(8):1079-1082.
9. Saadah OI, Zacharin M, O'Callaghan A, Oliver MR, Catto-Smith AG: **Effect of gluten-free diet and adherence on growth and diabetic control in diabetics with coeliac disease**. *ArchDisChild* 2004, **89**(9):871-876.
10. Kaspers S, Kordonouri O, Schober E, Grabert M, Hauffa BP, Holl RW: **Anthropometry, metabolic control, and thyroid autoimmunity in type 1 diabetes with celiac disease: A multicenter survey**. *JPediatr* 2004, **145**(6):790-795.
11. Mohn A, Cerruto M, Iafusco D, Prisco F, Tumini S, Stoppoloni O, Chiarelli F: **Celiac disease in children and adolescents with type I diabetes: importance of hypoglycemia**. *JPediatrGastroenterolNutr* 2001, **32**(1):37-40.
12. Westman E, Ambler GR, Royle M, Peat J, Chan A: **Children with coeliac disease and insulin dependent diabetes mellitus - Growth, diabetes control and dietary intake**. *Journal of Pediatric Endocrinology & Metabolism* 1999, **12**(3):433-442.
